# Phase 1b study of a small molecule antagonist of human chemokine (C-C motif) receptor 2 (PF-04136309) in combination with nab-paclitaxel/gemcitabine in first-line treatment of metastatic pancreatic ductal adenocarcinoma

**DOI:** 10.1007/s10637-019-00830-3

**Published:** 2019-07-12

**Authors:** Marcus Noel, Eileen M. O’Reilly, Brian M. Wolpin, David P. Ryan, Andrea J. Bullock, Carolyn D. Britten, David C. Linehan, Brian A. Belt, Eric C. Gamelin, Bishu Ganguly, Donghua Yin, Tenshang Joh, Ira A. Jacobs, Carrie T. Taylor, Maeve A. Lowery

**Affiliations:** 1 Department of Medicine, Division of Hematology/Oncology, University of Rochester Medical Center School of Medicine & Dentistry, Rochester, NY USA; 2 Department of Medicine, Memorial Sloan Kettering Cancer Center, Weill Cornell Medical College, New York, NY USA; 3 Department of Medical Oncology, Dana-Farber Cancer Institute, Boston, MA USA; 4 MGH Cancer Center, Division of Hematogy-Oncology, Massachusetts General Hospital, Boston, MA USA; 5 Division of Hematology/Oncology, Beth Israel Deaconess Medical Center, Boston, MA USA; 6 Division of Hematology/Oncology, Medical University of South Carolina, Charleston, SC USA; 7 Department of Surgery, University of Rochester Medical Center School of Medicine & Dentistry, Rochester, NY USA; 8 Department of Surgery, University of Rochester, Rochester, NY USA; 9 Early Oncology Development and Clinical Research, Pfizer Inc, 219 East 42nd Street, New York, NY 10017 USA; 10 Lyell Immunopharma Inc, Palo Alto, CA USA; 11 Trinity St James’s Cancer Institute, Trinity College Dublin, Dublin, Ireland

**Keywords:** CCR2 inhibitor, Pancreatic cancer, Immuno-oncology, Tumor-infiltrating macrophages, Tumor-infiltrating cells

## Abstract

*Background* In pancreatic ductal adenocarcinoma (PDAC), the chemokine (C-C motif) ligand 2 (CCL2)/chemokine (C-C motif) receptor 2 (CCR2) axis plays a key role in immunosuppressive properties of the tumor microenvironment, patient prognosis, and chemoresistance. This phase Ib study assessed the effects of the orally administered CCR2 inhibitor PF-04136309 in combination with nab-paclitaxel and gemcitabine in patients with previously untreated metastatic PDAC. *Methods* Patients received PF-04136309 twice daily (BID) continuously plus nab-paclitaxel (125 mg/m^2^) and gemcitabine (1000 mg/m^2^) administered on days 1, 8, and 15 of each 28-day cycle. The primary objectives were to evaluate safety and tolerability, characterize dose-limiting toxicities (DLTs), and determine the recommended phase II dose (RP2D) of PF-04136309. *Results* In all, 21 patients received PF-04136309 at a starting dose of 500 mg or 750 mg BID. The RP2D was identified to be 500 mg BID. Of 17 patients treated at the 500 mg BID starting dose, three (17.6%) experienced a total of four DLTs, including grade 3 dysesthesia, diarrhea, and hypokalemia and one event of grade 4 hypoxia. *Relative to the small number of patients (n = 21), a high incidence (24%) of pulmonary toxicity was observed in this study.* The objective response rate for 21 patients was 23.8% (95% confidence interval: 8.2–47.2%). Levels of CD14 + CCR2+ inflammatory monocytes (IM) decreased in the peripheral blood, but did not accumulate in the bone marrow. *Conclusions* PF-04136309 in combination with nab-paclitaxel plus gemcitabine had a safety profile that raises concern for synergistic pulmonary toxicity and did not show an efficacy signal above nab-paclitaxel and gemcitabine. ClinicalTrials.gov identifier: NCT02732938.

**Electronic supplementary material:**

The online version of this article (10.1007/s10637-019-00830-3) contains supplementary material, which is available to authorized users.

## Introduction

Metastatic pancreatic ductal adenocarcinoma (mPDAC) is a lethal disease with a median survival duration of less than 1 year [1–4]. While two combination regimens, FOLFIRINOX (*folinic* acid, fluorouracil, irinotecan, and oxaliplatin) and nab-paclitaxel/gemcitabine are used as standard therapies, the median survival associated with these regimens is less than a year, hence the need to seek other novel therapeutic approaches [5]. Progress in basic and translational immunology has confirmed the importance of controlling the immune system in cancer progression and in its treatment, and has renewed an interest in immune-based therapy for various cancers, including PDAC.

The main cellular components contributing to the immunosuppressive microenvironment include myeloid-derived suppressor cells (MDSCs), tumor associated macrophages (TAMs), mast cells, and T-regulatory cells (Tregs) [6, 7]. MDSCs comprise a heterogeneous population of immature cells of myeloid lineage that are considered to be key in orchestrating the suppressive tumor microenvironment. MDSC prevalence is increased in the peripheral blood and in the tumor microenvironment of patients with solid tumors, including PDAC [8]. In solid tumors, the number of circulating MDSCs significantly correlates with clinical state and metastatic tumor burden [9] and, in mice, reduction of MDSCs by inhibition [10] or deletion [11, 12] of factors that promote MDSC expansion has been shown to improve antitumor immune response [10], reduce primary and metastatic tumor progression [11], and abolish the tumor-promoting activity of MDSCs [11]. The pharmacologic modulation of MDSCs and prevention of their appearance or infiltration in solid tumors represent potential novel and innovative therapeutic strategies in cancer [10, 13–18].

In murine models of pancreatic cancer, it has been shown that MDSCs are upregulated in the tumor-bearing host, promote tumor growth, and suppress antitumor immunity [8]. The chemokine (C-C motif) ligand 2 (CCL2)/chemokine (C-C motif) receptor 2 (CCR2) signaling axis contributes to tumor progression through CCR2-mediated MDSC recruitment and/or accumulation [19–21]. PF-04136309, an orally administered CCR2 inhibitor, could block CCR2-mediated signal transduction, chemotaxis, and CCL2 binding in human monocytes and human whole blood. In addition, tumor-bearing wild-type mice treated with a CCR2 inhibitor demonstrated a significant decrease in liver metastasis compared with vehicle or gemcitabine-only treated mice [8]. These results suggest that CCR2 is a promising therapeutic target in PDAC, a condition associated with a marked upregulation of MDSCs in the tumor microenvironment in both mouse models and patients.

Previously, a phase Ib study demonstrated the CCR2 inhibitor PF-04136309 in combination with FOLFIRINOX significantly increased the proportion of patients achieving partial response (PR) compared to that anticipated with FOLFIRINOX alone [22]. The study also demonstrated the clinical activity of PF-04136309 correlated with an accumulation of CCR2+ inflammatory monocytes (IM) in the bone marrow, reduced levels of IM in peripheral blood, and decreased TAM in tumors. These encouraging results prompted the current study, which assessed the efficacy, safety, and tolerability, as well as the pharmacokinetics (PK) and pharmacodynamics, of PF-04136309 combined with nab-paclitaxel/gemcitabine in patients with mPDAC.

## Methods

### Study design

This was a multicenter phase Ib dose-finding study in the first-line treatment of patients with mPDAC. The study was open label and patients received prespecified doses of PF-04136309 in combination with nab-paclitaxel/gemcitabine. PF-04136309 was supplied as a formulated 125-mg tablet and given orally twice daily (BID) in 28-day cycles. Nab-paclitaxel (125 mg/m^2^) plus gemcitabine (1000 mg/m^2^) was administered in 28-day cycles by intravenous infusion over 30–40 min on days 1, 8, and 15 of each cycle, followed by 1 week off treatment.

In the dose-finding phase, a cohort of four patients was initially enrolled to receive the PF-04136309 starting dose of 750 mg BID in combination with nab-paclitaxel/gemcitabine in 28-day cycles. Observed toxicities in those patients led to a PF-04136309 dose reduction to 500 mg BID. Following the established safety observed in these four patients treated through the first cycle at 500 mg BID, the cohort was expanded with an additional 12 patients treated at this dose level to establish 500 mg BID as the recommended phase II dose (RP2D) of PF-04136309 in combination with nab-paclitaxel/gemcitabine. Although the phase II portion of the protocol was terminated by the sponsor, the development pathway for PF-04136309 is still under review.

The hypothetical mechanisms of action of PF-04136309 were explored by analysis of biopsies, bone marrow aspirates, and peripheral blood (pre- and post-treatment during the study). Serial blood samples were collected from patients to determine the multiple-dose PK of PF-04136309 given in combination with nab-paclitaxel/gemcitabine.

### Patient selection

Eligible patients were males and females ≥18 years of age, with histologically or cytologically proven diagnosis of mPDAC who had provided a baseline tumor sample at registration. Patients had not received previous radiotherapy, surgery, chemotherapy, or investigational therapy for the treatment of metastatic disease and had a life expectancy ≥12 weeks. Patients with Eastern Cooperative Oncology Group performance status (ECOG PS) 0 or 1 and adequate bone marrow, renal, and liver function were included. Patients with known symptomatic brain metastases requiring steroids or who had prior therapy with modulators of monocyte or TAM function in metastatic setting were ineligible to participate.

### Objectives

The primary objectives were to evaluate the safety and tolerability of PF-04136309 in combination with nab-paclitaxel/gemcitabine, to characterize the dose-limiting toxicities (DLTs), and determine the RP2D of PF-04136309. Secondary objectives included assessment of PF-04136309 PK analysis and ex vivo inhibition of CCL2-induced extracellular signal-regulated kinase phosphorylation (pERK) as a measure of target engagement. Exploratory objectives included evaluation of CCL2 levels in peripheral blood and the prevalence of IM, TAM, and other relevant immune cells in the peripheral blood, bone marrow, and core needle biopsy of metastases or fine-needle aspirate primary tumor tissue.

### Statistical methods

The modified intent-to-treat (mITT) population was defined as all patients who had received at least one dose of study medication and had measurable disease at baseline assessment (within 28 days prior to study entry). The mITT population was assessed for antitumor response.

### Safety

Safety assessments included collection of adverse events (AEs), serious AEs (SAEs), vital signs and physical examination, electrocardiogram (12-lead), laboratory assessments, including pregnancy test, and verification of concomitant treatments.

A patient was considered as DLT-evaluable if the patient experienced a DLT or if otherwise, in the absence of a DLT, the patient received at least 85% of the planned doses of each study drug in the first 28-day cycle. DLTs were defined as any of the following events occurring in the first cycle of treatment (days 1 through 28) and attributed (i.e., judged to be at least possibly related) to the combination of PF-04136309 plus nab-paclitaxel/gemcitabine, where relationship with the combination could not be ruled out. DLTs were hematologic events of grade 4 neutropenia lasting >5 days; febrile neutropenia; grade ≥ 3 neutropenic infection; grade ≥ 3 thrombocytopenia with grade ≥ 2 bleeding; grade 4 thrombocytopenia; and nonhematologic events of grade 3 toxicities. Exceptions included nausea and vomiting responding to prophylaxis and/or treatment and lasting <7 days from each chemotherapy infusion period; diarrhea responding to treatment and lasting <7 days; grade 3 fatigue lasting <7 days; grade 3 QT interval corrected for heart rate prolongation (>500 msec) persisting after correction of any reversible causes; and/or grade 3 aspartate aminotransferase (AST) and/or alanine aminotransferase (ALT) increase lasting ≤7 days. All grade 4 toxicities and a delay of >2 weeks in receiving the next scheduled cycle due to persistent treatment-related toxicities were considered DLTs. AEs meeting DLT criteria in the dose-expansion phase included grade 3 events of diarrhea, hypokalemia, and dysesthesia and grade 4 hypoxia. Treatment for these events followed the DLT defined by guidelines used during the dose-finding phase and did not change the identification of the RP2D in the study.

AEs were graded according to the National Cancer Institute Common Terminology Criteria for Adverse Events, version 4.03.

### Efficacy

Objective response rate (ORR) was defined as the proportion of patients with confirmed complete response (CR) or confirmed PR according to *Response Evaluation Criteria In Solid Tumors, version 1.1*, relative to all enrolled patients who had baseline measurable disease. Confirmed responses were those that persisted on repeat imaging ≥4 weeks after initial documentation of response. Indeterminate responses were those with no documented progression and absence of proper assessment of target lesions.

Patients without on-study radiographic tumor re-evaluation and those who died, progressed, or dropped out for any reason prior to reaching a CR or PR were counted as nonresponders in the assessment of ORR. The ORR, CR, and PR point estimates for each treatment arm were provided along with the corresponding two-sided 95% confidence intervals (CIs) using an exact method.

Imaging for tumor assessments included computed tomography (CT) or MRI scans of the chest, abdomen, and pelvis; brain CT or MRI scan for patients with known or suspected brain metastases; and bone scan and/or bone X-rays for patients with known or suspected bone metastases.

### Pharmacokinetic and pharmacodynamic assessment

Blood samples sufficient to provide ≥1 mL of plasma were collected for measurement of PF-04136309 concentrations. Plasma PF-04136309 concentrations were quantified with a validated liquid chromatography–mass spectrometry method. PF-04136309 concentration–time data from cycle 1 day 15 were analyzed using noncompartmental methods to estimate PK parameters.

The levels of CD14 + CCR2+ IM or other immune-cell phenotypes in the samples from the core needle biopsy, fine-needle aspirate from primary tumor tissue, bone marrow, and peripheral blood were assessed by flow cytometry. CCL2 levels were determined using whole blood plasma by immunoassay using a luminex-based method. The ex vivo inhibition of CCL2-induced pERK by PF-04136309 was measured in whole blood using flow cytometry. Percentages of CD4+, CD8+, and CD4 + FoxP3+ T cells within CD45+ populations were evaluated by flow cytometry in paired fresh biopsy samples from three patients. Two patients provided both baseline and on-study biopsies, and one patient provided a baseline biopsy only.

## Results

### Patient characteristics

Twenty-one patients (nab-paclitaxel/gemcitabine plus: PF-04136309 750 mg BID [*n* = 4] or 500 mg BID [*n* = 17]) were treated and included in the PK and safety analyses (Table 1). All 21 patients discontinued from both the treatment phase and study phase. Patients had ECOG PS 0 or 1, except for one patient in the 500-mg BID group who had baseline ECOG PS 2 – this patient had ECOG PS 1 during the screening period.

**Table 1:** Patient characteristics

	PF-04136309 BID + nab-P/Gem (*N* = 21)^a^		
	750 mg *n* = 4	500 mg *n* = 17^a^	Total *n* = 21
Age, mean (range)	61.3 (50–73)	61.9 (46–79)	61.8 (46–79)
Males / Females, *n*	0 / 4	11 / 6	11 / 10
Race, *n* (%)			
White	4 (100.0)	15 (88.2)	19 (90.5)
Black	0	1 (5.9)	1 (4.8)
Asian	0	1 (5.9)	1 (4.8)
ECOG PS			
0	3 (75.0)	8 (47.1)	11 (52.4)
1	1 (25.0)	8 (47.1)	9 (42.9)
2	0	1 (5.9)	1 (4.8)
Site of metastatic disease, *n* (%)			
Liver	2 (50.0)	14 (82.4)	16 (76.2)
Lung	1 (25.0)	6 (35.3)	7 (33.3)
Lymph node–Other	1 (25.0)	4 (23.5)	5 (23.8)
Lymph node–Supraclavicular	0	1 (5.9)	1 (4.8)
Peritoneum	0	2 (11.8)	1 (4.8)
Other	4 (100.0)	14 (82.4)	18 (85.7)
CA19.9 (U/mL) at baseline			
*n*	3	16	19
Mean	30197.33	15544.02	17857.70
Range	25.0–87160.0	0.5–112193.0	0.5–112193.0
Assigned to treatment, *n*			
Treated	4 (100)	17 (100)	21 (100)
Discontinued	4 (100)	17 (100)	21 (100)
DLT, *n* (%)^b^	1 (25)	3 (17.6)	4 (19.0)^c^

First subject–first visit: May 4, 2016; Last subject–first visit: September 15, 2017

^a^Twenty-two patients (*n* = 4 and 18 in the PF-04136309 750 mg BID + nab-paclitaxel/gemcitabine and PF-04136309 500 mg BID + nab-paclitaxel/gemcitabine groups, respectively) were assigned to study treatment, but one patient from the 500-mg BID group withdrew consent and did not receive study treatment

^b^DLT observation periods that occur in the first cycle of treatment (days 1 through 28) and are attributed (i.e., judged to be at least possibly related) to the combination of PF-04136309 plus nab-paclitaxel/gemcitabine where relationship with the combination cannot be ruled out. DLTs are classified according to CTCAE version 4.03. A patient is classified as DLT evaluable if he/she experiences a DLT or a DLT is absent but patient receives 85% of the planned doses of each study drug in the first 28-day cycle

^c^5 DLTs were reported in four patients

Abbreviations: *CA-19.9* cancer antigen 19.9, *CTCAE* Common Terminology Criteria for Adverse Events, *DLT* dose-limiting toxicities, *ECOG PS* Eastern Cooperative Oncology Group performance status, *nab-P/Gem* nab-paclitaxel/gemcitabine

### Safety

Previous clinical studies demonstrated that PF-04136309 was generally safe and well tolerated after a single oral dose of up to 1000 mg, and after repeated oral administration up to 500 mg BID as a single agent or in combination with FOLFIRINOX [22]. Repeated dosing with >500 mg BID was previously untested. In this study, repeated PF-04136309 dosing of 750 mg BID was evaluated for the first time, in combination with nab-paclitaxel/gemcitabine.

In the 750-mg BID group (*n* = 4), the most frequently reported (≥75%) all-causality treatment-emergent AEs (TEAEs) were nausea and fatigue (*n* = 4 each [100.0%]) and leukopenia, neutropenia, constipation, vomiting, ALT increase, alopecia, and rash (*n* = 3 each [75.0%]) (Table 2). Each of the four (100.0%) patients had at least one grade 3 TEAE: the most frequently reported (≥50%) events were leukopenia and neutropenia (*n* = 3 each [75.0%]) (Supplementary Table S1). No patient experienced grade 4 TEAE or treatment-related death. The most frequently reported PF-04136309-related TEAE (≥30%) was rash (*n* = 3 [75.0%]).

**Table 2:** Summary of TEAEs^a^ in >25% patients in any treatment group (all-causality, all cycles) – safety analysis set

	Grade 1 *n* (%)	Grade 2 *n* (%)	Grade 3 *n* (%)	Grade 4 *n* (%)	Grade 5 *n* (%)	Total *N* (%)
	PF-04136309 750 mg BID + nab-P/Gem (*n* = 4)					
Any AE	0	0	4 (100.0)	0	0	4 (100.0)
Anemia	0	2 (50.0)	0	0	0	2 (50.0)
Leukopenia	0	0	3 (75.0)	0	0	3 (75.0)
Neutropenia	0	0	3 (75.0)	0	0	3 (75.0)
Thrombocytopenia	1 (25.0)	1 (25.0)	0	0	0	2 (50.0)
Constipation	2 (50.0)	1 (25.0)	0	0	0	3 (75.0)
Nausea	3 (75.0)	0	1 (25.0)	0	0	4 (100.0)
Vomiting	2 (50.0)	1 (25.0)	0	0	0	3 (75.0)
Chills	2 (50.0)	0	0	0	0	2 (50.0)
Fatigue	3 (75.0)	1 (25.0)	0	0	0	4 (100.0)
Malaise	2 (50.0)	0	0	0	0	2 (50.0)
Pyrexia	1 (25.0)	1 (25.0)	0	0	0	2 (50.0)
Cellulitis	0	1 (25.0)	1 (25.0)	0	0	2 (50.0)
ALT increase	0	2 (50.0)	1 (25.0)	0	0	3 (75.0)
Appetite decrease	1 (25.0)	0	1 (25.0)	0	0	2 (50.0)
Pain in extremity	1 (25.0)	1 (25.0)	0	0	0	2 (50.0)
Insomnia	1 (25.0)	1 (25.0)	0	0	0	2 (50.0)
Alopecia	2 (50.0)	1 (25.0)	0	0	0	3 (75.0)
Rash	2 (50.0)	1 (25.0)	0	0	0	3 (75.0)
	PF-04136309 500 mg BID + nab-P/Gem (*n* = 17)					
Any AE	0	1 (5.9)	12 (70.6)	3 (17.6)	1 (5.9)	17 (100.0)
Anemia	2 (11.8)	5 (29.4)	4 (23.5)	0	0	11 (64.7)
Lymphopenia	0	0	5 (29.4)	0	0	5 (29.4)
Abdominal pain	0	3 (17.6)	3 (17.6)	0	0	6 (35.3)
Constipation	5 (29.4)	2 (11.8)	0	0	0	7 (41.2)
Diarrhea	4 (23.5)	2 (11.8)	1 (5.9)	0	0	7 (41.2)
Nausea	12 (70.6)	2 (11.8)	0	0	0	14 (82.4)
Vomiting	6 (35.3)	1 (5.9)	0	0	0	7 (41.2)
Fatigue	4 (23.5)	4 (23.5)	5 (29.4)	0	0	13 (76.5)
Edema, peripheral	7 (41.2)	1 (5.9)	0	0	0	8 (47.1)
Pyrexia	7 (41.2)	5 (29.4)	0	0	0	12 (70.6)
ALT increase	1 (5.9)	2 (11.8)	3 (17.6)	1 (5.9)	0	7 (41.2)
AST increase	0	1 (5.9)	5 (29.4)	0	0	6 (35.3)
Blood alkaline phosphatase	1 (5.9)	4 (23.5)	2 (11.8)	0	0	7 (41.2)
Weight decreased	5 (29.4)	4 (23.5)	0	0	0	9 (52.9)
Appetite decrease	5 (29.4)	0	1 (5.9)	0	0	6 (35.3)
Hyperglycemia	2 (11.8)	3 (17.6)	3 (17.6)	0	0	8 (47.1)
Headache	4 (23.5)	1 (5.9)	0	0	0	5 (29.4)
Neuropathy peripheral	8 (47.1)	1 (5.9)	0	0	0	9 (52.9)
Insomnia	7 (41.2)	1 (5.9)	0	0	0	8 (47.1)
Alopecia	7 (41.2)	3 (17.6)	0	0	0	10 (58.8)
Rash	7 (41.2)	1 (5.9)	0	0	0	8 (47.1)

^a^Maximum grade per Common Terminology Criteria for Adverse Events [CTCAE]

Abbreviations: *AE* adverse event, *ALT* alanine aminotransferase, *AST* aspartate aminotransferase, *BID* twice daily, *N* number of evaluable patients, *n* number of patients in the category, *nab-P/Gem* nab-paclitaxel/gemcitabine, *TEAE* treatment-emergent adverse event

Of the four treated patients in the 750-mg BID group, one (25.0%) experienced a DLT of grade 3 cognitive disorder that occurred on cycle 1 day 7 and resolved on the same day; the patient was disorientated, had loss of memory, and presented with an acute onset of speech difficulties, which lasted several hours and required a visit to the emergency department. An ischemic stroke was ruled out for this patient and there was no previous neurologic history of stroke. This DLT was considered to be related to treatment with PF-04136309 and resulted in a dose reduction to 250 mg BID, which continued until discontinuation from treatment phase. The remaining three patients did not experience DLTs, but all experienced AEs (grade 1–2 events of peripheral sensory neuropathy, rash, rash maculopapular, or cellulitis) that resulted in dose reduction of PF-04136309. To allow better tolerability, the PF-04136309 dose was reduced to 500 mg BID in combination with nab-paclitaxel/gemcitabine for the subsequent cohort of patients.

In the 500-mg BID group (*n* = 17), the most frequently reported (≥60%) all-causality TEAEs were nausea (*n* = 14 [82.4%]), fatigue (*n* = 13 [76.5%]), pyrexia (*n* = 12 [70.6%]), and anemia (*n* = 11 [64.7%]) (Table 2). Twelve (70.6%) patients had at least one grade 3 TEAE; the most frequently reported (≥20%) events were lymphopenia, fatigue, and increased AST (*n* = 5 each [29.4%]) and anemia (*n* = 4 [23.5%]) (Table 2). Three patients (17.6%) had at least one grade 4 TEAE, including increased ALT (Table 2), decreased neutrophil count and white blood cell count, and hypoxia (*n* = 1 each [5.9%]) (data not shown). The most frequently reported PF-04136309-related TEAE (≥30%) was rash (*n* = 6 [35.3%]).

Of the 17 treated patients in the 500-mg BID group, three (17.6%) experienced a total of four DLTs: one patient with two DLTs of grade 3 diarrhea and hypokalemia, one patient with one DLT of grade 3 dysesthesia, and one patient with one DLT of grade 4 hypoxia. Grade 3 pneumonitis was reported as a late-onset DLT. Additionally, one patient experienced grade 5 SAE of pneumonia (Supplementary Table S2), which was considered unrelated to PF-04136309, but determined by the sponsor to be possibly related to gemcitabine and nab-paclitaxel.

Eleven deaths occurred in total, all in the PF-04136309 500-mg BID group. The main cause of death (10 out of 11) was disease under study, all of which occurred after the SAE reporting period (i.e., after 28 days following the last dose of study treatment). The one death that occurred during the SAE reporting period was due to study treatment toxicity (the aforementioned grade 5 pneumonia). The patient was admitted to an intensive care unit due to hypoxic respiratory failure. A right-sided central line was placed for administration of medications, which resulted in a right-sided pneumothorax. Although diagnosed as pneumonia, pathogens were not cultured. Despite the fact that the patient was provided with antibiotics, supplemental oxygen, and supportive care, the patient continued to desaturate and required higher ventilation setting and respiratory care, and subsequently developed acute renal failure with rising levels of creatinine. The patient died approximately 7 days following hospital admission.

Overall, 14 (66.7%) of the 21 treated patients experienced SAEs (Supplementary Table S2) and seven (33.3%) patients experienced treatment-related SAEs (attributed to at least one of the three exposure drugs [PF-04136309, nab-paclitaxel, or gemcitabine]) (Supplementary Table S3). Most of the SAEs were in one patient each. Four patients had SAEs that led to permanent discontinuations of study treatment. Of note, three patients experienced SAEs of pneumonitis, all of which were considered treatment-related and resulted in permanent discontinuations of study treatment (Supplementary Table S3). Two patients who were permanently discontinued from treatment experienced a grade 4 SAE of hypoxia and a grade 2 non-SAE of ALT increase, respectively, both of which were considered treatment-related. Five patients were identified with an acute pulmonary AE that may have been attributable to the combination of nab-paclitaxel, gemcitabine, and PF-04136309 (Supplementary Table S3). The grade 4 AEs of decreased neutrophil counts, decreased white blood cell counts, and increased ALT, observed in one patient each in the 500-mg BID group, were all considered unrelated to study treatment. There were no clinically significant changes in laboratory tests consistent with a relationship to study drug. There were no clinically significant changes in vital signs data consistent with a relationship to study drug and no consistent pattern in findings on physical examinations. The RP2D for PF-04136309 in combination with nab-paclitaxel/gemcitabine was determined to be 500 mg BID.

### Efficacy

**Fig. 1:**
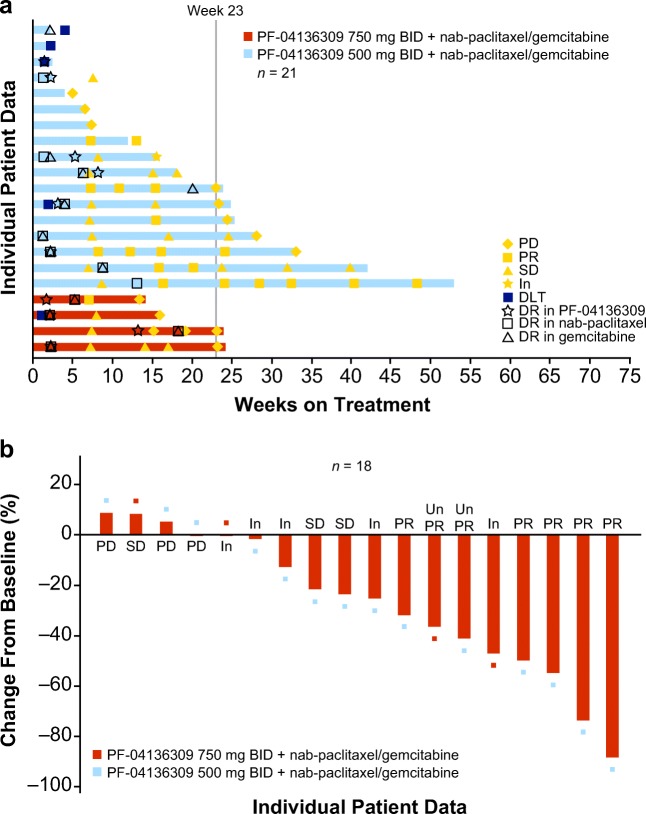
**Clinical response. a** Bar plot for duration of treatment by dose level (including response, dose reduction, and DLT). mITT population was analyzed. Only the first time of dose reduction is presented. Each bar represents one patient in the study. Duration was calculated as follows: (last dose date – first dose date +1) / 7. **b** Waterfall plot of tumor size percent change data. mITT population was analyzed. Largest decrease or smallest increase represents best response to treatment. Only patients with target lesions at baseline and at least one post-baseline target lesion based on investigator assessment per RECIST version 1.1 are included (*n* = 18). Abbreviations: *BID* twice daily, *DLT* dose-limiting toxicity, *DR* dose reduction, *In* indeterminate, *mITT* modified intent-to-treat, *PD* progressive disease, *PR* partial response, *RECIST* Response Evaluation Criteria in Solid Tumors, *SD* stable/no response, *Un PR* unconfirmed partial response

All 21 treated patients had measurable disease at baseline assessment and were included in the mITT population. None of the 21 treated patients achieved CR (Fig. 1a and Supplementary Table S4). Five patients (all in the 500-mg BID group) showed a best overall response of PR. Of the four treated patients in the 750-mg BID group, the best overall response observed was unconfirmed PR in one (25.0%) patient, stable disease in one (25.0%) patient, and was indeterminate in two (50.0%) patients (Fig. 1 and Supplementary Table S4). Of the 17 treated patients in the 500-mg BID group, the best overall response observed was PR in five (29.4%) patients, unconfirmed PR in one (5.9%) patient, stable disease in two (11.8%) patients, objective progression in three (17.6%) patients, early death in one (5.9%) patient, and was indeterminate in five (29.4%) patients (Fig. 1a and Supplementary Table S4). For all 21 patients who received treatment, the ORR was 23.8% (95% exact CI, 8.2–47.2%). In the 500 mg BID group (*n* = 17) the ORR was 29.4% (95% exact CI, 10.3–56.0%) (Supplementary Table S4). Overall survival was not evaluated in this study. Due to the study being terminated prematurely, only 2 of the 17 patients in the 500 mg group and 1 of the 4 patients in the 750 mg group had progression events and the rest were censored. The median progression-free survival (mPFS) for the 500 mg group was 5.3 months, but due to the censoring and the small number of patients with progression events, mPFS is not an appropriate estimate in this regard.

### PK assessment

PF-04136309 was quickly absorbed following oral administration, with a median time to maximum plasma concentration (T_max_) of 1.42 h at the 500-mg BID dosage. The observed values of area under the plasma concentration–time (AUC) profile within the dose interval tau (AUC_tau_; 10,600 and 15,700 ng·hr/mL) and maximum plasma concentration (C_max_; 2950 and 3390 ng/mL) values were higher in the 750-mg BID group (*n* = 2) compared with the mean AUC_tau_ (5873 ng·hr/mL) and mean C_max_ (1276 ng/mL) in the 500-mg BID group. At 500-mg BID dosing (*n* = 13), steady-state PK parameters for PF-04136309 were associated with a moderate interpatient variability, with a coefficient of variation of 44%, 57%, and 30% for C_max_, C_min_, and AUC_tau_, respectively. There was no apparent correlation between cycle 1 steady-state plasma exposure and ORR or the occurrence of DLTs.

### Pharmacodynamic assessment

**Fig. 2:**
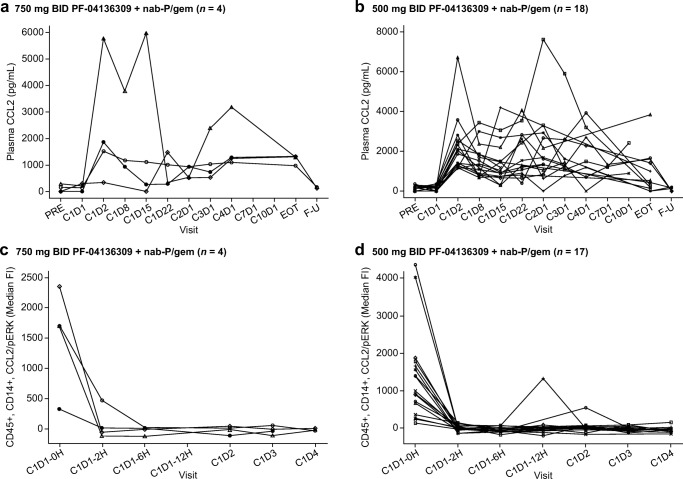
**Effect of PF-04136309 in combination with nab-paclitaxel/gemcitabine on the CCL2 pathway. a** and **b** Plasma CCL2 levels of individuals were examined by an immunoassay using a luminex-based method. **c** and **d** Individual plots of CCL2-induced pERK by treatment group. Target engagement was measured by an ex vivo CCL2-induced pERK assay**. a** and **c** treatment group: 750 mg BID PF-04136309 + nab-paclitaxel/gemcitabine. **b** and **d** treatment group: 500 mg BID PF-04136309 + nab-paclitaxel/gemcitabine. Each symbol represents individual patient. Abbreviations: *BID* twice daily, *C* cycle, *CCL2* the chemokine (C-C motif) ligand 2, *D* day, *EOT* end of treatment, *Fl* fluorescence intensity, *F-U* follow-up, *H* hour, *nab-P/gem* nab-paclitaxel/gemcitabine, *pERK* phosphorylated extracellular signal regulated kinase phosphorylation, *PRE* before treatment

**Fig. 3:**
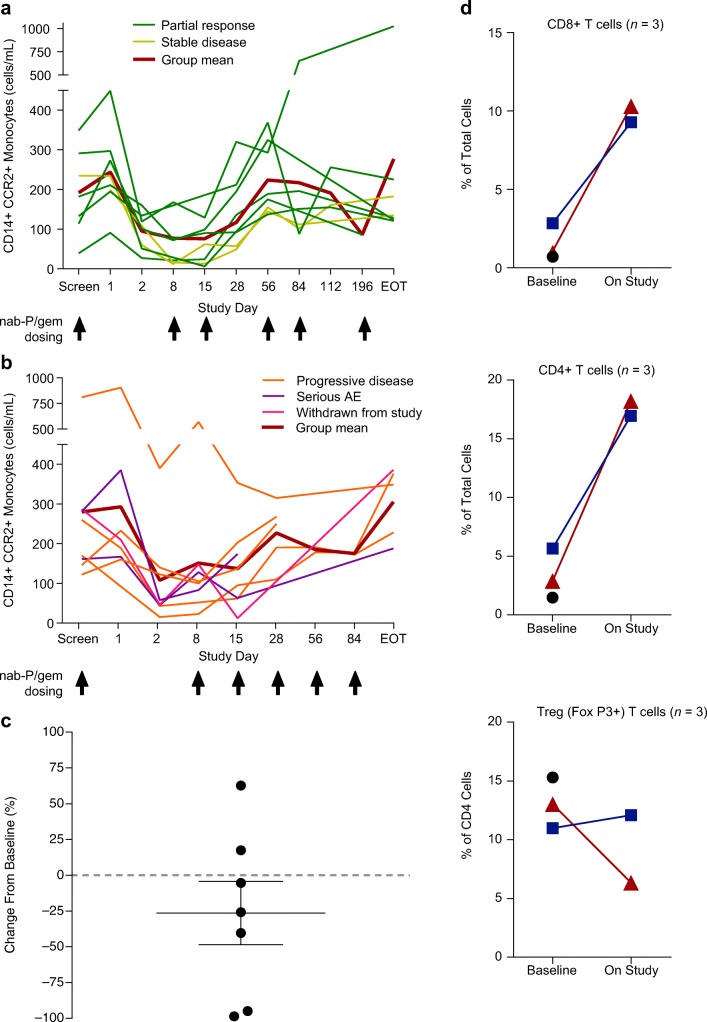
**Changes of immune cell levels in the peripheral blood, bone marrow, or tumor after dosing with PF-04136309 in combination with nab-paclitaxel/gemcitabine.** Changes of CD14+ CCR2+ monocytes in patients with (**a**) best response of stable disease or partial response or (**b**) patients with progressive disease, serious adverse event, or withdrawn from study. **c** Percentage change of CCR2+ monocytes in the bone marrow. Bar represents SEM. **d** Percentage of CD8+, CD4+, and CD4 + FoxP3+ T cells in biopsy samples at baseline and week 6. Each symbol represents individual patient. Abbreviations: *AE* adverse event, *CCR2* chemokine (C-C motif) receptor 2, *EOT* end of treatment, *nab-P/gem* nab-paclitaxel/gemcitabine, *SEM* standard error of mean; *Treg* regulatory T cell

An increase in CCL2 levels (Fig. 2a, b) and drop in pERK (Fig. 2c, d) was observed in most patients over the course of study at both the 500 mg (*n* = 17) and 750 mg (*n* = 4) BID doses of PF-04136309. Nearly all patients exhibited a drop in absolute counts of CD14 + CCR2+ IM in peripheral blood from baseline (study day 1 pre-dose) to study day 2 and the level was sustained through study day 15 (Fig. 3a, b). Patients in the 500-mg BID group with a best response of stable disease or PR exhibited a drop in monocyte counts between day 1 pre-dose and day 2 (Fig. 3a). A similar, but less consistent pattern was observed in patients with progressive disease, SAEs, or in those who withdrew from the study (Fig. 3b). An accumulation of CCR2 + CD14+ monocytes in the bone marrow was not observed at week 6 post dosing compared with baseline in patients treated with PF-04136309 500 mg BID (Fig. 3c). Two patients in the 500-mg BID group exhibited an increase in CD4+ and CD8+ cells within CD45+ populations measured in fresh biopsy tumor samples (Fig. 3d). CCR2+ TAM levels also fell in the aforementioned two patients in the 500-mg BID group (~6% to ~1% and ~4% to 3%, respectively; data not shown). It should be noted that on-treatment biopsies were not mandatory and very few patients provided consent for this procedure.

## Discussion

mPDAC is a lethal disease with poor 5-year survival and is projected to be the second leading cause of cancer death by 2020 in the United States [23, 24]. Although both FOLFIRINOX and gemcitabine combined with nab-paclitaxel improve patient survival and disease response compared with single-agent gemcitabine, there is an immunosuppressive tumor microenvironment directed in part by the CCL2/CCR2 axis. Since the degree of therapeutic resistance with metastatic spread affects the lethality of aggressive cancers, understanding and targeting the mechanisms that are responsible for chemoresistance is critical to improving therapeutic outcomes. Immunologic targeting, in particular, is considered a key to effective treatment of this refractory disease [25, 26].

In a prior phase II study, patients with previously untreated advanced pancreatic cancer dosed with 125 mg/m^2^ nab-paclitaxel plus 1000 mg/m^2^ gemcitabine on days 1, 8, and 15 every 28 days reported grade ≥ 3 TEAEs of neutropenia, leukopenia, thrombocytopenia, and anemia [27], with a median progression-free survival and overall survival of 7.9 months and 12.2 months, respectively. In the current study, we observed similar grade ≥ 3 hematologic TEAEs (except thrombocytopenia) with PF-04136309 500 mg BID in combination with nab-paclitaxel/gemcitabine and following the same dosing and treatment schedule as the previously reported phase II study [27].

In addition to those hematologic TEAEs, we observed three patients with grade 3 pneumonitis, one patient with grade 4 hypoxia, and one patient with grade 5 pneumonia among the 21 patients in the current study. The patient with grade 5 pneumonia received PF-04136309 500 mg BID in cycle 1 through day 16, and was hospitalized due to pneumonia 6 days later. It is important to note that both nab-paclitaxel and gemcitabine have a known association with pulmonary toxicity, mainly pneumonitis, both alone and in combination (synergistically). An observed rate of pneumonitis in patients treated with gemcitabine was approximately 1% and was elevated up to 4% when combined with nab-paclitaxel [3], leading to a high level of morbidity. Furthermore, advanced-stage disease, smoking, and alcohol consumption, and possibly underlying lung disease, can be potential risk factors of gemcitabine-related pneumonitis [28]. Infections, in particular, are frequent complications in patients with malignancies. Although relevant laboratory information was not available, bone-marrow suppression, as a predisposing factor for infection as well as dyspnea and pneumonitis, is a common side effect of nab-paclitaxel and gemcitabine. The patient with grade 5 pneumonia was a smoker and thus had an additional predisposing factor for respiratory tract infections. Nevertheless, we observed a relatively high incidence (24%) of pulmonary toxicity in this study, whereas no pulmonary events have been reported in patients administered PF-04136309 as a single agent in non-oncology studies (*N* = 178: 76 healthy volunteers and 102 patients) and in 39 patients with advanced PDAC treated with PF-04136309 in combination with FOLFIRINOX [22]. Some of the pulmonary toxicity observed in this study may have been caused by the combination between PF-04136309 and gemcitabine with nab-paclitaxel.

Multiple measures may need to be undertaken in order to further understand these pulmonary events. The clinical outcome would likely depend on the particular macrophage populations involved (immune regulatory vs. pro-immune) and characteristics of the local tissue environment, since the mechanism of action of PF-04136309 is the inhibition of the trafficking of IMs from the bone marrow to the tumor, resulting in the depletion of TAMs from tumor microenvironment, enhancing antitumor immunity [22]. On the other hand, depletion of macrophages from healthy tissues could theoretically increase the probability of autoimmune-mediated inflammation or, alternatively, the probability of infection, potentially elevating the risk of gemcitabine-related pneumonitis. Further validation by a larger study is necessary to clarify mechanisms of pulmonary toxicity, which may be caused by the combination of nab-paclitaxel, gemcitabine, and PF-04136309.

Previous phase II and III studies in patients with PDAC demonstrated that a regimen of nab-paclitaxel plus gemcitabine had tolerable adverse effects with antitumor activity [27] and improved patients’ survival and response rate [3] compared with gemcitabine alone. PF-04136398 in combination with FOLFIRINOX was shown to be well-tolerated and demonstrated clinical activity in patients with borderline resectable and locally advanced pancreatic cancer [22]. In the present study, although the efficacy of PF-04136309 in combination with nab-paclitaxel/gemcitabine could not be evaluated adequately in a small patient population, based on all 21 patients, an ORR of 23.8% was observed; the ORR for the 500 mg BID treatment group (*n* = 17) was 29.4%.

The hypothesized mechanism of action of PF-04136309 is the inhibition of CCL2-induced trafficking of IM from the bone marrow to the tumor. Three components of this proof of mechanism are the depletion of TAM from the tumor, a decrease of CD14 + CCR2+ IM in the peripheral blood, and the accumulation of CD14 + CCR2+ IM in bone marrow. Although we observed a drop of CD14 + CCR2+ IM in peripheral blood, a decrease of CCR2+ TAM in the tumor was only observed for two patients and is inadequate to support a definitive conclusion about this component of the mechanism of action. Further, an accumulation of CCR2 + CD14+ IM in the bone marrow did not take place at week 6 post dose compared with baseline, unlike a previous study examining the combination of PF-04136309 with FOLFIRINOX [22]. The lack of accumulation of IM in the bone marrow can be explained in part by the previous observation that gemcitabine treatment resulted in persistence of IM in the peripheral blood of patients [8], thus possibly counterbalancing the effect of PF-04136309 with regard to IM maintenance in the bone marrow.

A recent study that analyzed patient samples and mice with established orthotopic tumors demonstrated that patients with non-metastatic PDAC administered a CCR2 inhibitor showed a compensatory influx of C-X-C motif chemokine receptor 2 [CXCR2] + tumor-associated neutrophils (TAN), an increase that correlates with poor prognosis in PDAC [29]. Targeting CCR2+ TAM and CXCR2+ TAN in combination caused influx of both CD8+ and CD4+ T cells in the tumor microenvironment, improving antitumor immunity and reducing tumor burden [29]. In the present study, one patient who received PF-04136309 had an increase in programmed cell death protein 1 (PD-1) + CD4+ and PD-1 + CD8+ cells in the tumor biopsy sample (data not shown), suggesting PF-04136309 also modulated PD-1 immune checkpoint in mPDAC. These results imply CCR2 inhibition reprogrammed the immunosuppressive tumor microenvironment and that tumor-induced immune plasticity in response to treatment with CCR inhibitors may be responsible for therapeutic resistance.

Although our data are limited by the nonrandomized design and small sample size, some clinical activity was observed with the combination of PF-04136309 and nab-paclitaxel/gemcitabine; nonetheless the combination had a safety profile that raises concern for synergistic pulmonary toxicity in patients with mPDAC. Inhibition of CCR2 by treatment with PF-04136309 in the presence of nab-paclitaxel/gemcitabine resulted in a drop of IM in peripheral blood and tumor, but unexpectedly did not accumulate IM in the bone marrow, possibly due to compensatory activity by gemcitabine.

## Electronic supplementary material

ESM 1 (DOCX 26 kb)
