# Supplementary material for: Phase 1b study of a small molecule antagonist of human chemokine (C-C motif) receptor 2 (PF-04136309) in combination with nab-paclitaxel/gemcitabine in first-line treatment of metastatic pancreatic ductal adenocarcinoma
Source: Invest New Drugs. 2019 Jul 12;38(3):800–11. doi: 10.1007/s10637-019-00830-3 (PMC7211198; doi:10.1007/s10637-019-00830-3)
Supplement: Supplementary file 1 — (DOCX 26 kb) [file 10637_2019_830_MOESM1_ESM.docx]

Supplementary Table S1 Treatment-emergent, treatment-related adverse events, grade ≥3
(≥5% occurrence)

|  | **PF-04136309 BID Dose Group** | |  |
| --- | --- | --- | --- |
|  | **750 mg + nab-P/Gem *n* = 4** | **500 mg + nab-P/Gem  *n* = 17** | **Total *N* = 21** |
| Hematologic AE, *n* (%) | | | |
| Anemia | 0 | 4 (23.5) | 4 (19.0) |
| Leukopenia | 3 (75.0) | 2 (11.8) | 5 (23.8) |
| Neutropenia | 3 (75.0) | 2 (11.8) | 5 (23.8) |
| Lymphopenia | 1 (25.0) | 4 (23.5) | 5 (23.8) |
| Nonhematologic AE, *n* (%) | | | |
| ALT increase | 1 (25.0) | 3 (17.6) | 4 (19.0) |
| AST increase | 1 (25.0) | 3 (17.6) | 4 (19.0) |
| Fatigue | 0 | 4 (23.5) | 4 (19.0) |
| Pneumonitis | 0 | 3 (17.6) | 3 (17.6) |

500 mg PF-04136309 addition had no impact on nab-P and Gem dose intensity.

Abbreviations: *AE* adverse event, *ALT* alanine aminotransferase, *AST* aspartate aminotransferase, *BID* twice daily, *nab-P/Gem* nab-paclitaxel/gemcitabine

**Supplementary Table S2** Summary of treatment-emergent serious adverse events* (all-causality, all cycles) – safety analysis set

| **Adverse Event, *n* (%)** | **Grade 1** | **Grade 2** | **Grade 3** | **Grade 4** | **Grade 5** | **Total** |
| --- | --- | --- | --- | --- | --- | --- |
|  | **750 mg BID PF-04136309 + nab-P/Gem (*N* = 4)** | | | | | |
| Any SAE | 0 | 1 (25.0) | 2 (50.0) | 0 | 0 | 3 (75.0) |
| Gastroesophageal reflux disease | 0 | 0 | 1 (25.0) | 0 | 0 | 1 (25.0) |
| Cellulitis | 0 | 0 | 1 (25.0) | 0 | 0 | 1 (25.0) |
| Appetite decrease | 0 | 0 | 1 (25.0) | 0 | 0 | 1 (25.0) |
| Cognitive disorder | 0 | 0 | 1 (25.0) |  | 0 | 1 (25.0) |
| Rash | 0 | 1 (25.0) | 0 | 0 | 0 | 1 (25.0) |
|  | **500 mg BID PF-04136309 + nab-P/Gem (*N* = 17)** | | | | | |
| Any SAE | 1 (5.9) | 1 (5.9) | 7 (41.2) | 1 (5.9) | 1 (5.9) | 11 (64.7) |
| Episcleritis | 0 | 1 (5.9) | 0 | 0 | 0 | 1 (5.9) |
| Abdominal pain | 0 | 0 | 2 (11.8) | 0 | 0 | 2 (11.8) |
| Small intestinal obstruction | 0 | 0 | 1 (5.9) | 0 | 0 | 1 (5.9) |
| Chest pain | 0 | 0 | 1 (5.9) | 0 | 0 | 1 (5.9) |
| Pyrexia | 1 (5.9) | 1 (5.9) | 0 | 0 | 0 | 2 (11.8) |
| Biliary sepsis | 0 | 0 | 1 (5.9) | 0 | 0 | 1 (5.9) |
| Klebsiella bacteremia | 0 | 0 | 1 (5.9) | 0 | 0 | 1 (5.9) |
| Liver abscess | 0 | 0 | 1 (5.9) | 0 | 0 | 1 (5.9) |
| Pneumonia | 0 | 0 | 0 | 0 | 1 (5.9) | 1 (5.9) |
| Sepsis | 0 | 0 | 1 (5.9) | 0 | 0 | 1 (5.9) |
| Musculoskeletal pain | 0 | 0 | 1 (5.9) | 0 | 0 | 1 (5.9) |
| Neck pain | 0 | 0 | 1 (5.9) | 0 | 0 | 1 (5.9) |
| Dyspnea | 0 | 0 | 1 (5.9) | 0 | 0 | 1 (5.9) |
| Hypoxia | 0 | 0 | 0 | 1 (5.9) | 0 | 1 (5.9) |
| Pneumonitis | 0 | 0 | 3 (17.6) | 0 | 0 | 3 (17.6) |

*By maximum grade per Common Terminology Criteria for Adverse Events.

Abbreviations: *ALT* alanine aminotransferase, *AST* aspartate aminotransferase, *BID* twice daily,
*N* number of evaluable patients, *n* number of patients in the category, *nab-P/Gem* nab-paclitaxel/ gemcitabine, *SAE* serious adverse event

**Supplementary Table S3** Summary of treatment-emergent serious adverse events^a^ (treatment-related, all cycles) – safety analysis set

| **Adverse Event, n (%)** | **Grade 1** | **Grade 2** | **Grade 3** | **Grade 4** | **Grade 5** | **Total** |
| --- | --- | --- | --- | --- | --- | --- |
|  | **750 mg BID PF-04136309 + nab-P/Gem (*N* = 4)** | | | | | |
| Any SAE | 0 | 1 (25.0) | 1 (25.0) | 0 | 0 | 2 (50.0) |
| Cognitive disorder | 0 | 0 | 1 (25.0) | 0 | 0 | 1 (25.0) |
| Rash | 0 | 1 (25.0) | 0 | 0 | 0 | 1 (25.0) |
|  | **500 mg BID PF-04136309 + nab-P/Gem (*N* = 17)** | | | | | |
| Any SAE | 0 | 0 | 3 (17.6) | 1 (5.9) | 1 (5.9) | 5 (29.4) |
| Pyrexia | 0 | 1 (5.9) | 0 | 0 | 0 | 1 (5.9) |
| Pneumonia | 0 | 0 | 0 | 0 | 1 (5.9) | 1 (5.9) |
| Hypoxia | 0 | 0 | 0 | 1 (5.9) | 0 | 1 (5.9) |
| Pneumonitis | 0 | 0 | 3 (17.6) | 0 | 0 | 3 (17.6) |
| ^a^By maximum grade per Common Terminology Criteria for Adverse Events.  *BID* twice daily, *N* number of evaluable patients, *n* number of patients in the category,  *nab-P/Gem* nab-paclitaxel/gemcitabine, *SAE* serious adverse event | | | | | | |

|  | **PF-04136309 BID Dose Group** | |
| --- | --- | --- |
| ***n* (%)** | **750 mg + nab-P/Gem  *N* = 4** | **500 mg + nab-P/Gem  *N* = 17** |
| Complete response | 0 | 0 |
| Partial response | 0 | 5 (29.4) |
| Unconfirmed complete response | 0 | 0 |
| Unconfirmed partial response | 1 (25.0) | 1 (5.9) |
| Stable/no response | 1 (25.0) | 2 (11.8) |
| Objective progression | 0 | 3 (17.6) |
| Early death | 0 | 1 (5.9) |
| Indeterminate | 2 (50.0) | 5 (29.4) |
| Objective response rate (95% exact CI^a^) | 0 (0.0–60.2) | 5 (29.4) (10.3–56.0) |
| ^a^Using exact method based on binominal distribution.  Abbreviations: *BID* twice daily, *CI* confidence interval, *mITT* modified intent-to-treat, *N* number of evaluable patients, *n* number of patients in the category, *nab-P/Gem* nab-paclitaxel/gemcitabine | | |

**Supplementary Table S4** Summary of best overall response – mITT set
